# Plasma membrane proteomics reveals mechanism of low potassium tolerance in two contrasting maize genotypes

**DOI:** 10.3389/fpls.2026.1957989

**Published:** 2026-09-24

**Authors:** Ming Zhong, Shuisen Chen, Xudong Yang, Xiaojie Wang, Silu Li, Dian Jiao, Fei Zou, Jing Li, Yanye Ruan, Zhifu Guo, Pedro Garcia-Caparros

**Affiliations:** 1 College of Biological Science and Biotechnology, Shenyang Agricultural University, Shenyang, China; 2 Maize Research Institute, Hulunbuir Academy of Agricultural and Animal Husbandry Sciences, Zhalantun, China; 3 Higher Engineering School, University of Almeria, Almeria, Spain

**Keywords:** K efficiency, low K stress, maize, plasma, proteomics, roots vitality

## Abstract

Potassium (K) deficiency is a major constraint on plant growth and crop productivity. To elucidate the genotypic mechanisms underlying maize adaptation to K deficiency, we comprehensively compared the physiological responses and 8-plex iTRAQ (4 biological replicates) based plasma membrane (PM) proteomes of two maize genotypes with contrasting K efficiency. Under low-K conditions, the K-efficient genotype (K-ef) maintained greater growth and root vitality and exhibited significantly higher K concentration and accumulation than the K inefficient genotype (K-inef). Quantitative plasma membrane (PM) proteomic analysis revealed 52 differentially accumulated proteins (DAPs) in K-ef (29 upregulated and 23 downregulated) and 49 DAPs in K-inef (38 upregulated, and 11 downregulated). These DAPs were involved in transport, signal transduction, lipid metabolism, cellular structure, and redox/defense. Integrating the physiological and proteomic data revealed that K-ef may adopt an “energy-saving” strategy by prioritizing K^+^ transport, redox defense, and Ca^2+^ signaling, whereas K-inef falls into an energy-wasting strategy and indiscriminate stress response. Our study provides valuable insights into physiological and molecular mechanisms underlying low K stress in maize roots and providing a basis to further study K nutrition in other crop species.

## 1 Introduction

Potassium (K) is a critical macronutrient required for plant growth, governing processes such as osmotic balance, enzyme activation, stomatal regulation, and stress resilience (Cui and Tcherkez, 2021; Cao et al., 2025). Despite its abundance in soils, most of the soil K exists in an unavailable state, which only 0.1%-0.2% of K can be directly absorbed by plants through roots (Hafsi et al., 2014). Consequently, K insufficiency in soil leads to reduced nutrient use efficiency and yield instability (Hussain et al., 2021). K deficiency significantly inhibits root development and overall plant growth, and is a prevalent and significant constraint on crop production (Liang et al., 2025). Agricultural production faces a widespread challenge of soil K deficiency, affecting approximately 20% of the world’s arable land (Brownlie et al., 2024), while nearly 40% of cultivated soils in China are deficient in available K (Wu et al., 2013). Furthermore, crops utilize only about 50% of applied K fertilizer attributable to low K use efficiency (Britto and Kronzucker, 2008). This challenge is particularly pronounced in China, where maize production requires substantial K fertilizer inputs, yet the K uptake/utilization efficiency of maize cultivars remains relatively low (Zhang et al., 2023). Nevertheless, plants have evolved sophisticated adaptive response mechanisms to K deficiency. Therefore, it is urgent to reveal the mechanism of crop tolerance to K deficiency and to develop crops with high K uptake and use efficiency.

The plasma membrane (PM) is not only a selective barrier that protects the cell from the extracellular environment, but also essential for maintaining cellular homeostasis (Vasconcelos-Cardoso et al., 2022). PM proteins play pivotal roles in diverse biological processes, including signal transduction, ions transportation, cell wall biosynthesis and defense against biotic and abiotic stresses (Liu et al., 2015). Approximately 30% of genes in eukaryotic genomes are predicted to encode membrane proteins, and this proportion is even higher in plants with large genomes (Wallin and von Heijne, 1998). In plant roots, PM proteins are indispensable for coordinating nutrient acquisition, maintaining ion homeostasis, and mediating adaptive responses to environmental fluctuations. Consequently, PM proteomics is one of the key approaches to explore membrane proteins and elucidating their biological functions. However, the lower abundance and hydrophobic nature of PM proteins pose a huge challenge for PM proteins preparation and identification in proteomics. In plant, the PM constitutes only about 5-20% of all cellular membranes in a cell (Szymanski et al., 2013). Therefore, the purification of high-quality PM is challenging but important to analyze plant responses to environmental stress. Among the available methods, an aqueous two-phase partitioning in polyethylene glycol and dextran has been frequently used for PM isolation and purification, in which the purity of PM vesicles could up to 95% (Alexandersson et al., 2004).

Maize (Zea mays), a staple crop for food and feed, is particularly vulnerable to low-K stress. Maize exhibits substantial genetic variation in K use efficiency, with contrasting genotypes showing divergent responses to low-K stress (Al-Rawi et al., 2023). In the past decades, several genes associated with K absorption and transport in maize have been identified. For instance, overexpressing K channel 1(*ZMK1*, a member of Shaker family) and *ZmHAK1/5* (high-affinity K transporter) in maize roots enhances K uptake and promotes maize growth (Han et al., 2021; Qin et al., 2019). In addition, transcriptomic analysis of maize seedling roots revealed the key genes including 17 associated with K signaling, transcription factors, and transporters (Guo et al., 2023). However, although these studies have advanced our understanding of K-deficiency responses at the transcriptional level, the dynamic changes in plasma membrane (PM) proteins, which directly mediate nutrient transport and signal transduction, remain largely unexplored. In the present study, root PM of two maize genotypes with contrasting K efficiency under low K stress were isolated and purified using an aqueous two-phase partitioning system. Quantitative proteomic analysis based on isobaric tags for relative and absolute quantification (iTRAQ) was subsequently performed to identify PM proteins related to K deficiency. The identification of alterations in the PM proteins in response to K deficiency in maize roots would provide information to better understand the mechanism by which plants can adapt to environmental stresses. The present study not only reveals the core regulatory modules conferring low K tolerance in maize seedlings but also provide crucial insights for breeding stress-resilient crops.

## 2 Material and methods

### 2.1 Plant growth

Two low K tolerance contrasting maize inbred lines, K-efficient maize genotype (K-ef, tolerant to low K stress) and K-inefficient maize genotype (K-inef, sensitive to low K stress), were used in the present study. The maize seeds were surface-sterilized with7% sodium hypochlorite (NaClO) solution. The seeds were soaked in distilled water for 24 h and then sown in moist fine sand for two weeks under controlled growth conditions. The culture conditions were as follow: 300 - 320 μmol·m^-2^·s^-1^ flux density, 16 h light (22°C)/8 h dark (20°C) photoperiod and 60% relative humidity. After two weeks, uniform seedlings were transferred to a modified Hoagland-Arnon nutrient solution and cultured for an additional week. The nutrient solution was renewed every 4 days and contained 2.00 mM Ca(NO_3_)_2_, 1.25 mM KCl, 1.00 mM Mg_2_SO_4_, 0.50 mM NH_4_H_2_PO_4_, 0.10 mM FeSO_4_-EDTA, 115 μM H_3_BO_3_, 22.5 μM MnCl_2_, 0.16 μM CuSO_4_, 0.75 μM ZnSO_4_ and 0.182 μM (NH_4_)_6_Mo_7_O_24_. Three-week-old seedlings were randomly divided into two treatment groups. Half of the seedlings were transferred to a modified Hoagland–Arnon nutrient solution containing 0.10 mM KCl to impose low-potassium stress (−K), while the remaining seedlings were maintained in the complete Hoagland–Arnon nutrient solution containing 1.25 mM KCl as the control (+K). To maintain ionic balance between the two treatments, KCl was replaced with an equimolar concentration of NaCl in the low-K nutrient solution. Before transferring, the roots were gently rinsed with deionized water to remove residual nutrient solution. Both treatment groups were grown under the same controlled environmental conditions described above. Root and shoot tissues were harvested separately after 3 days of K treatment, immediately frozen in liquid nitrogen, and stored at −80 °C until further analysis.

### 2.2 Measurements of biomass and K content

Roots and shoots were harvested separately after K stress for 3 d. After washing with deionized water, the surface moisture of roots and shoots was removed using absorbent paper. The fresh weight (FW) of each sample was immediately measured, wrapped in a kraft paper envelope and oven dried at 105°C for 15 min. Then, each sample was dried at 70 °C until a constant weight and the respective dry weight (DW) was measured. For K content assay, root or shoot (ca. 0.1 g, DW) was finely ground and digested using sulfuric acid and hydrogen peroxide. The root and shoot K concentrations were measured via flame photometry. The K accumulation of root and shoot was calculated by multiplying the K concentration by the corresponding dry weight (DW) of each sample.

### 2.3 Root morphology and root vitality assay

Root morphological traits, including total root length, average root diameter, root surface area and root volume, were determined using an EPSON V800 scanner (Epson, Japan). The scanned root images were analyzed with WinRHIZO software (Regent Instruments Inc., Quebec, Canada) to quantify the corresponding root morphological parameters.

Root vitality was determined using the triphenyltetrazolium chloride (TTC) reduction method as previously described by Xia et al. (2023). Briefly, root tip (ca. 0.5 g, FW) was incubated in a reaction mixture containing 5 mL 0.4% TTC and 5 mL 1/15 M phosphate buffer (pH 7.0) at 37°C for 1 h in the dark. Then, 2 mL of 1 M sulfuric acid was immediately added to terminate the reaction. The root samples were then dried with a filter paper and homogenized with quartz sand in 4 mL of ethylacetate. The homogenate was filtered into a clean centrifuge tube, and the residue was extracted two times more with ethyl acetate. Finally, the extracted solution was adjusted to a final volume of 10 mL with ethylacetate. The absorbance of the extract was measured at 485 nm. Root vitality was expressed as the amount of TTC reduced per unit fresh weight per hour and calculated using the following equation: (μg·TTC g^-1^ FW·h^-1^) = tetrazolium reduction amount (μg)/[root fresh weight (g) × incubation time (h)].

### 2.4 K^+^ absorption of roots in maize

To determine root K^+^ uptake kinetics, three-week-old seedlings were first transferred to a K-free nutrient solution (0 mM K) and subjected to K starvation for 3 days. After the starvation treatment, lateral roots were removed, and root tips were excised 5 cm from the apex. Approximately 0.1 g (fresh weight, FW) of root tips was immersed in 200 mL of uptake solution containing 0.5 mM CaCl_2_ (pH 6.0) and the desired K^+^ concentration. Root K^+^ uptake was monitored using a K^+^-selective electrode connected to an automatic data acquisition system, and K^+^ concentrations in the uptake solution were continuously recorded for 96 min. Uptake assays were performed using initial K^+^ concentrations of 0.15, 0.10, and 0.05 mM, respectively.

### 2.5 Plasma membrane isolated and PM proteins identified

Fresh root tissue (ca. 50 g) collected from maize seedlings was used for PM isolation. Plasma membranes were purified using an aqueous two-phase partitioning system consisting of 6.2% (w/w) dextran T500 and 6.2% (w/w) PEG3350. The purity of the isolated PM fraction was evaluated by measuring ATPase activity, as described previously (Chen et al., 2016).

Purified PM proteins were precipitated with pre-chilled acetone and the resulting protein pellet was washed by pre-cooled 80% ethanol. Protein digestion and iTRAQ labeling procedure was performed according to the manufacturer´s instructions for the 8-plex iTRAQ reagent kit (Applied Biosystems), as previously described in our study (Yang et al., 2026). Relative iTRAQ quantification was performed with ProteinPilot™ Software 5.0 (AB SCIEX). A strict unused confidence score > 1.3 was used. The identified proteins with matched peptides > 2, confidence > 95%, FDR (false discovery rate) < 1% were used to perform protein quantification. A two-tailed t-test was employed and a p-value was calculated for each protein. The proteins detected in at least three of the four biological replicates were used for further analysis. Proteins abundance with a 1.5-fold change (p < 0.05) were termed differentially abundant proteins (DAPs).

### 2.6 Bioinformatics analysis

TMHMM (https://services.healthtech.dtu.dk/services/TMHMM-2.0/) was used to predict the number of transmembrane helices of the identified PM proteins. STRING (version 12.0) (https://string-db.org) was used for protein-protein interaction analysis and KEGG (Kyoto Encyclopedia of Genes and Genomes) pathway annotations.

### 2.7 Statistical analysis

All physiological data were analyzed using SAS software (version 9.3; SAS Institute Inc., Cary, NC, USA). Analysis of variance (ANOVA) was performed using the PROC ANOVA procedure, and treatment means were compared using the least significant difference (LSD) test. Differences were considered statistically significant at p < 0.05. All data are presented as the mean ± standard deviation (SD) of three biological replicates.

## 3 Results

### 3.1 Effects of low K stress on biomass and K concentration

Low K stress significantly inhibited maize growth. Under low K stress for 3 d, both root and shoot dry weights were significantly reduced in the K-inefficient maize genotype (K-inef) (**Figure 1**). In contrast, no significant differences in root or shoot dry weights were observed in the K-efficient maize genotype (K-ef) in response to low K stress (**Figure 1**). These results indicate that low K stress affected the growth of K-inef more than K-ef.

**Figure 1:**
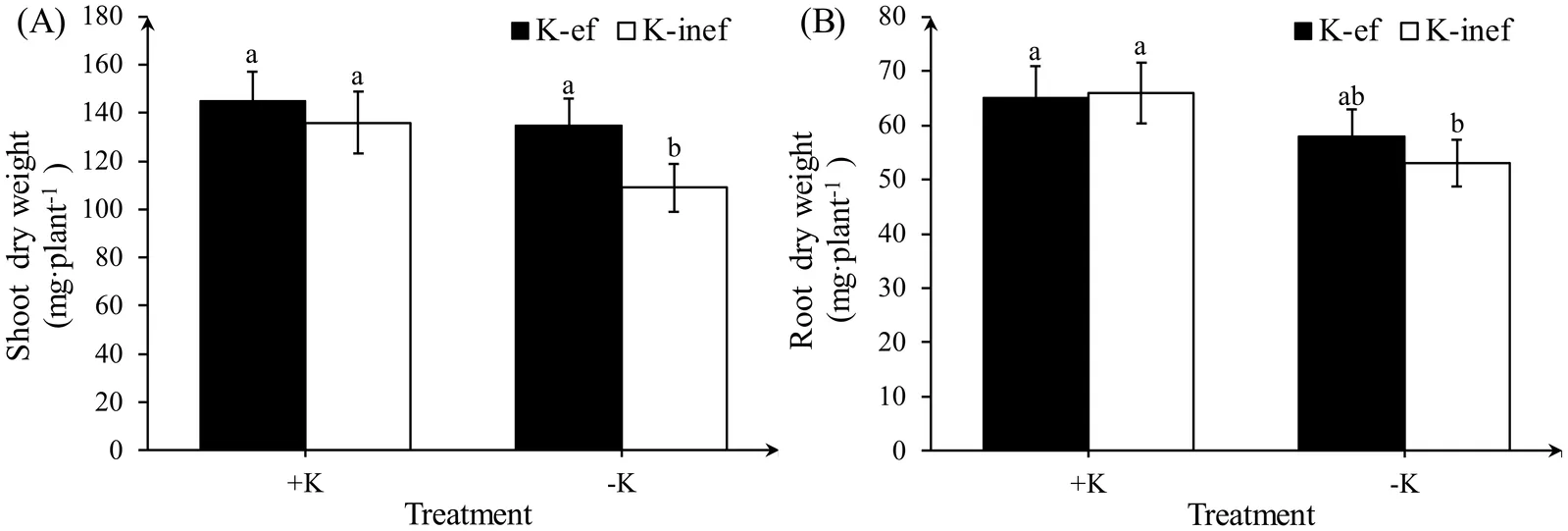
The effect of low K stress on shoot **(A)** and root **(B)** dry weight of maize genotypes with different K efficiency. The +K and -K represent the seedlings under high K (1.25 mM K) and low K (0.1 mM K) concentration respectively. Values represent the mean ± SD of three replicates. Bars with different letters show significant differences at p<0.05.

Under high K conditions, no significant differences in root or shoot K concentrations were observed between the two maize genotypes with contrasting K efficiency (**Figures 2A, B**). Low K treatment significantly decreased K concentrations in both roots and shoots of both genotypes. However, the concentrations of K in root and shoot in K-efficient maize genotype (K-ef) were significantly higher than that in K-inefficient maize genotype (K-inef) under low K stress (**Figures 2A, B**). A similar trend was observed for K accumulation. The K-efficient maize genotype (K-ef) showed significantly higher K accumulation than the K-inefficient maize genotype (K-inef) under low-K stress (**Figures 2C, D**). Specifically, shoot K accumulation in K-ef was 1.85-fold higher than that in K-inef (**Figure 2C**), while root K accumulation was 1.91-fold higher (**Figure 2D**). These results indicate that the K efficient maize genotype (K-ef) maintained a higher K absorption ability compared to the K inefficient maize genotype (K-inef) in response to low K stress.

**Figure 2:**
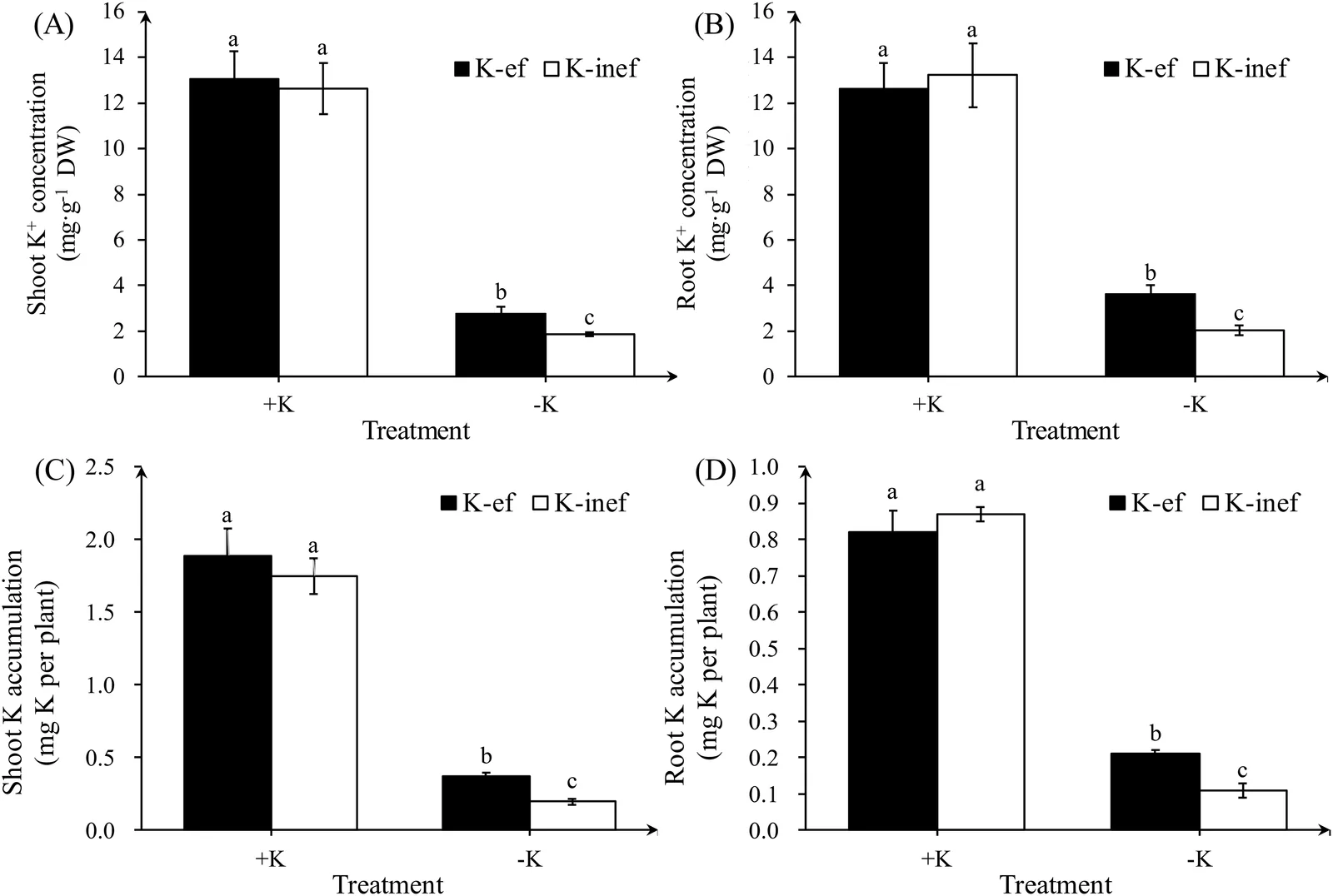
The effect of low K stress on shoot **(A)** and root **(B)** K content, shoot **(C)** and root **(D)** K accumulation of maize genotypes with different K efficiency. The +K and -K represent the seedlings under high K (1.25 mM K) and low K (0.1 mM K) concentration, respectively. Values represent the mean ± SD of three replicates. Bars with different letters show significant differences at p<0.05.

### 3.2 Effects of low K stress on morphology of maize seedlings

To investigate whether the difference in K efficiency between the two maize genotypes was associated with root morphology, root morphological traits in response to low K stress were evaluated. The total root length and total root surface area of K-inefficient maize genotype (K-inef) was slightly higher than that of K-efficient maize genotype (K-ef) in response to low K stress but without significant differences (**Figures 3A, C**). In contrast, the average root diameter and the total root volume of K-efficient maize genotype (K-ef) were significantly lower than that of K-inefficient maize genotype (K-inef) (**Figures 3B, D**). These results indicate that the higher K efficiency of K-ef was not due to a larger root architecture.

**Figure 3:**
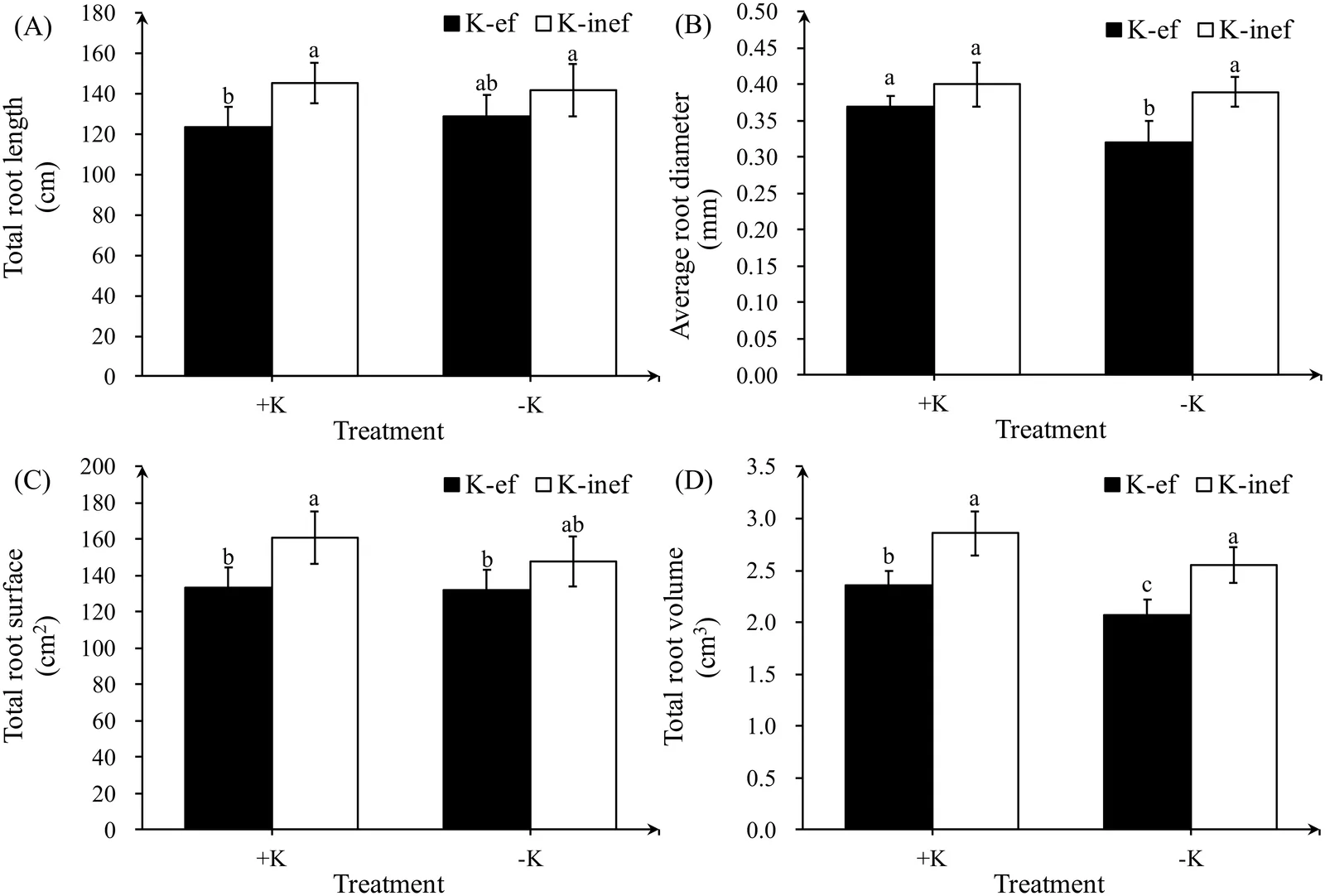
The effect of low K stress on the total root length **(A)**, average root diameter **(B)**, total root surface **(C)**, and total root volume **(D)** of maize genotypes with different K efficiency. The +K and -K represent the seedlings under high K (1.25 mM K) and low K (0.1 mM K) concentration, respectively. Values represent the mean ± SD of ten seedlings in each treatment. Bars with different letters show significant differences at p<0.05.

### 3.3 Effects of low K stress on root vitality

Root vitality refers to the overall functional capacity of the root system to sustain essential physiological and biochemical processes involved in plant growth, nutrient uptake, and stress resilience. Low K stress significantly impacted the growth and development of roots and significantly affected the health of roots, which was manifested by changes in root vitality (**Figure 4**). After 3 days of low K stress, the root vitalities of the two maize genotypes significantly declined (**Figure 4**). Under high K conditions, root vitality of the K-efficient maize genotype (K-ef) was significantly lower than that of the K-inefficient maize genotype (K-inef). In contrast, under low K stress, root vitality of the K-efficient maize genotype (K-ef) was significantly higher than that of the K-inefficient maize genotype (K-inef) (**Figure 4**). Compared with the corresponding high-K treatment, root vitalities of the seedlings under low K stress on the 3rd day decreased by 26.23% and 54.79% in K-ef and K-inef, respectively (**Figure 4**). These results indicate that the superior root vitality of the K-efficient maize genotype (K-ef) was specifically induced under K deficiency, enabling it to maintain better root physiological function and thereby greater adaptability to low-K environments.

**Figure 4:**
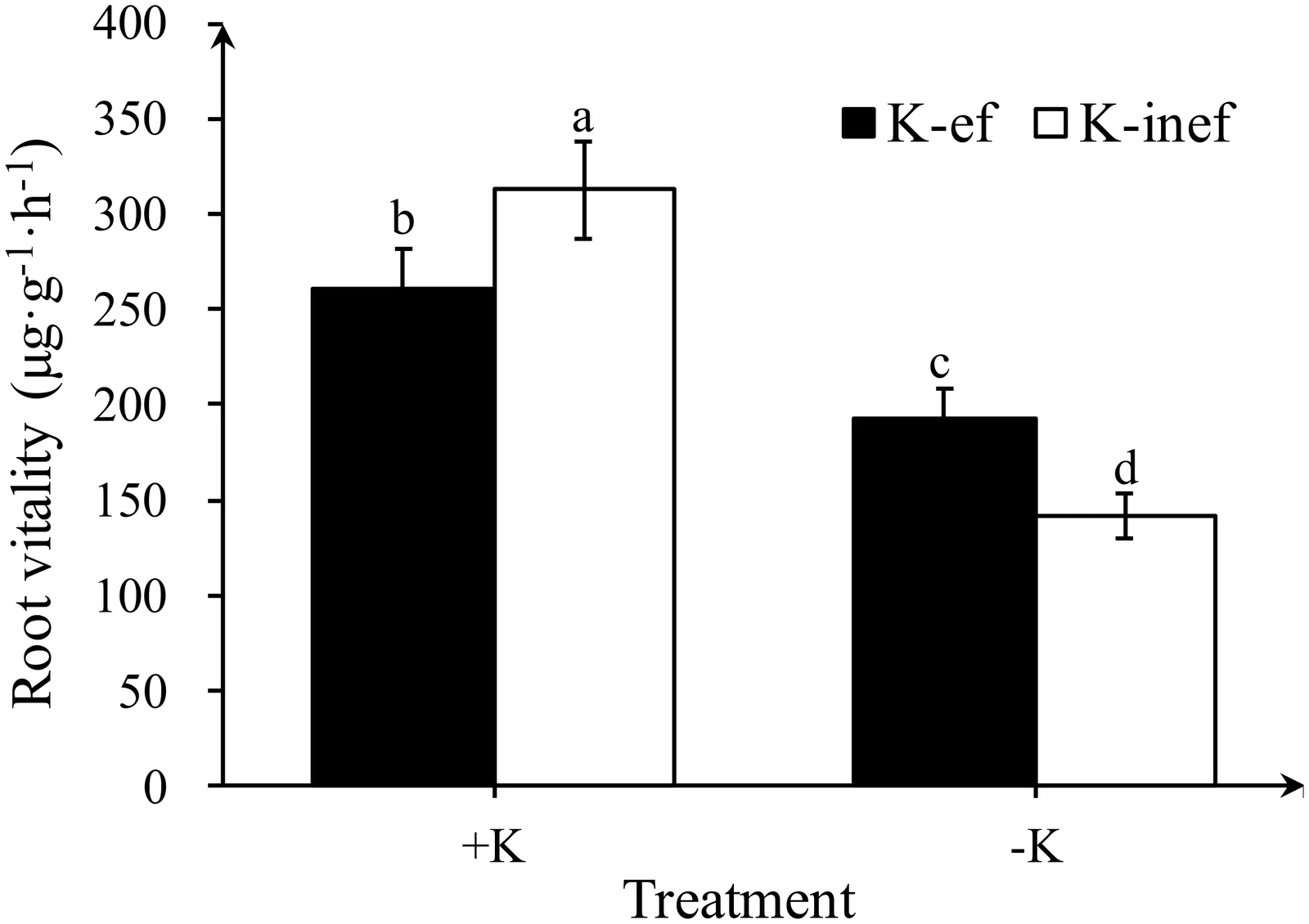
The effect of low K stress on root vitality of maize genotypes with different K efficiency. The +K and -K represent the seedling under high K (1.25 mM K) and low K (0.1 mM K) concentration, respectively. Values represent the mean ± SD of three replicates. Bars with different letters show significant differences at p<0.05.

### 3.4 Comparison between the K absorption of root in maize with different K efficiency

Under a 0.15 mM K solution, the K absorption of roots in K-ef and K-inef were 6.62 and 5.11 μmol K·g^−1^ FW respectively within the 96 min (**Figure 5**). The ratio of K absorption of K-ef to K-inef was 1.22. Under the 0.10 mM K solution, the K^+^ absorption of roots in K-ef and K-inef decreased to 5.33 and 3.00 μmol K·g^−1^ FW respectively within the 96 min (**Figure 5**), while the ratio of K absorption of K-ef to K-inef increased to 1.78. When the K concentration of the solution was 0.05 mM, the K absorption of roots in K-ef and K-inef were 2.34 and 1.02 μmol K·g^−1^ FW respectively within the 96 min (**Figure 5**). The ratio of K absorption of K-ef to K-inef was 2.29. These results indicate that the K efficient maize (K-ef) had a stronger K absorption capacity compared to the K inefficient maize (K-inef), especially under the lower K concentration.

**Figure 5:**
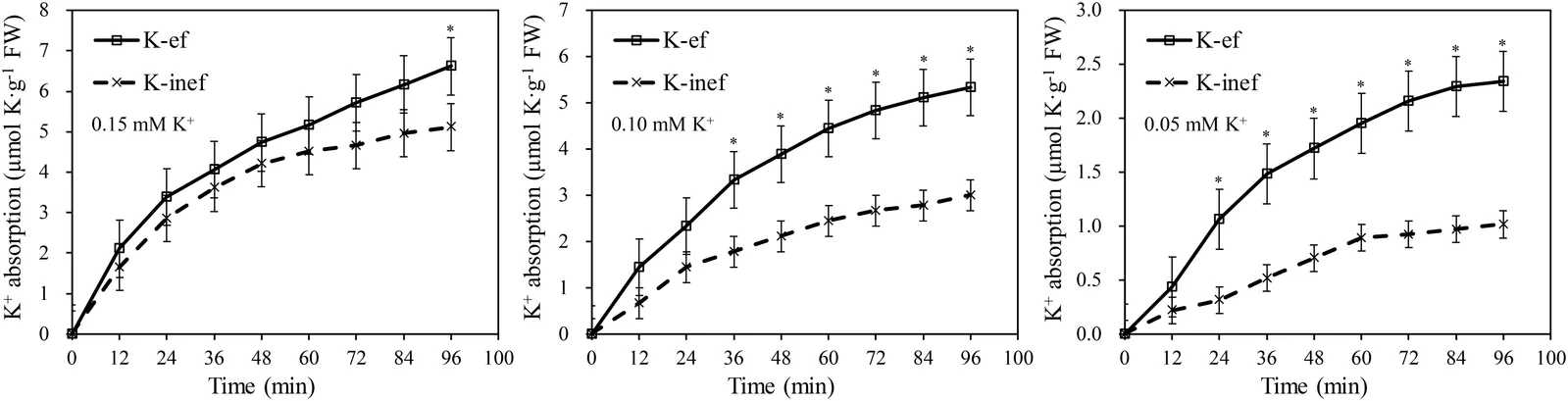
The K^+^ absorption of maize genotypes with different K efficiency under different K concentrations. Values represent the mean ± SD of three replicates. * means significant differences at p < 0.5 level.

### 3.5 PM proteins with differential abundance under K stress

Quantitative proteomic analysis was employed to detect PM proteins in maize root in response to low K stress. There were 52 DAPs in the K efficient maize genotype (K-ef) under low K stress, of which 29 DAPs increased in protein abundance and 23 DAPs decreased in protein abundance (**Table 1**). In the K inefficient maize genotype (K-inef), 49 DAPs were identified, of which 38 DAPs increased in protein abundance and 11 DAPs decreased in protein abundance in response to low K stress (**Table 2**). These DAPs were classified into five functional categories including transportation, signal transduction, lipid metabolism, cellular structure and redox and defense. There were 10 DAPs with same protein abundance changes in response to low K stress including 6 DAPs showing increased protein abundance and 4 DAPs decreased protein abundance, respectively (**Figure 6**). In contrast, three DAPs displayed opposite abundance patterns, being upregulated in K-inef but downregulated in K-ef (**Figure 6**). These results indicate that the two maize genotypes employ distinct molecular mechanisms to respond to K deficiency. Protein-protein interaction analysis serves as a crucial bridge connecting molecular mechanisms to system-level regulatory mechanisms. The PM DAPs of K-ef formed 2 pairs of interacting proteins and 1 interaction network. One protein pair comprised two upregulated proteins involved in redox and defense, whereas the other consisted of two downregulated transport-related proteins. The interaction network consisted of 3 DAPs of which 2 increased in protein abundance and 1 decreased in protein abundance (**Figure 7A**). In the protein-protein interaction of K-inef, 14 PM DAPs formed 2 pairs of interacting proteins and 2 interaction networks. One network consisted of 6 DAPs, of which 5 increased in protein abundance being involved in signal transduction and 1 decreased in protein abundance being involved in transportation. The second network consisted of 4 DAPs, of which 3 increased in protein abundance and 1 decreased in protein abundance (**Figure 7B**). Among the interacting protein pairs, one included the upregulated proteins peroxidase and fasciclin-like arabinogalactan protein 10, whereas the other consisted of two downregulated transport-related proteins. Under low-K stress, the K-efficient maize genotype (K-ef) showed a simple and functionally balanced protein–protein interaction network comprising mainly redox/defense and transport proteins. In contrast, the K-inefficient maize genotype (K-inef) formed a complex interaction network dominated by overactivated signal transduction modules, revealing distinct system-level regulatory strategies in response to K deficiency.

**Table 1:** List of the DAPs of K efficient maize genotype in response to low K stress.

No.	Protscore	Coverage (95%)	Peptides (95%)	Accession no.	Name	Fold change	THMs
Transportation							
1	16.82	22.4	16	B4F8Y4	Transmembrane 9 superfamily member	1.50 ± 0.12	10
2	6.05	7.0	4	W5U4T4	Potassium transporter	18.19 ± 1.22	13
3	6.78	24.5	5	B4F821	Syntaxin 23	50.04 ± 5.41	1
4	16.04	81.8	31	B4FPE4	V-type proton ATPase subunit G	1.75 ± 0.15	0
5	45.42	74.0	34	B4FMY6	V-type proton ATPase subunit C	2.02 ± 0.31	0
6	52.09	56.6	50	C0P4T2	Patellin-1	4.33 ± 0.51	0
7	66.49	54.2	47	B4FBC9	Patellin-1	5.28 ± 0.65	0
Signal transduction							
8	6.19	11.8	5	B8A0V6	non-specific serine/threonine protein kinase	10.95 ± 0.81	0
9	14.81	8.8	9	K7V594	non-specific serine/threonine protein kinase	1.59 ± 0.19	0
10	24.41	44.7	17	B6TXE2	Protein kinase domain-containing protein	1.57 ± 0.06	0
11	4.53	12.8	3	B6UHC3	Calmodulin	10.25 ± 1.11	0
12	13.15	35.0	10	C4JBJ3	Calmodulin-like protein 1	44.04 ± 3.75	0
13	2.42	2.9	4	Q9FV60	Calmodulin-binding protein MPCBP	39.46 ± 4.56	0
14	19.39	21.0	15	B6U1M5	Calcium ion binding protein	1.67 ± 0.15	9
15	2.32	21.3	6	B4FU46	Calcium-dependent lipid-binding (CaLB domain) family protein	44.45 ± 4.51	0
16	20.88	28.7	18	B4G0H5	Membrane-associated salt-inducible protein like	2.37 ± 0.17	0
17	66.55	92.2	130	B4FKM1	Guanine nucleotide-binding protein beta subunit-like protein	2.23 ± 0.13	0
18	61.37	95.8	138	B6SJ21	Guanine nucleotide-binding protein beta subunit-like protein	1.97 ± 0.21	0
19	45.26	78.5	48	B6SRI4	14-3-3-like protein	2.54 ± 0.15	0
20	7.16	17.7	4	B6TED9	14-3-3-like protein	2.83 ± 0.17	0
21	10.22	14.8	5	B6TW73	Diacylglycerol kinase	1.83 ± 0.12	0
22	6.10	8.5	4	C0PJK5	IQ-domain 3	2.14 ± 0.14	0
23	26.12	56.7	14	B4FUC4	Plasma membrane-associated cation-binding protein 1	3.19 ± 0.34	0
24	71.38	75.5	62	C4J6K9	Guanosine nucleotide diphosphate dissociation inhibitor	1.80 ± 0.11	0
25	3.07	7.5	2	B4FV79	Suppressor/enhancer of lin-12 protein 9	3.76 ± 0.26	1
26	11.62	58.6	9	B4FA79	EF-hand domain-containing protein	1.95 ± 0.17	0
Lipid metabolism							
27	46.04	80.7	31	Q49HE1	12-oxo-phytodienoic acid reductase	2.51 ± 0.09	0
28	4.41	4.8	3	A1XCI3	Lipoxygenase	1.77 ± 0.23	0
29	116.50	75.0	84	Q06XS2	Lipoxygenase	1.70 ± 0.18	0
30	78.34	81.5	100	B4FHT1	Annexin	1.59 ± 0.11	0
31	61.19	80.6	83	Q43863	Annexin	1.62 ± 0.18	0
Cellular structure							
32	19.77	39.8	12	B4FPG3	Hydroxyproline-rich glycoprotein family protein	2.20 ± 0.27	0
33	6.38	18.4	7	B6TC02	Beta-expansin 4	5.78 ± 0.34	1
34	30.11	77.6	41	K7W544	Putative carboxylesterase 2	1.62 ± 0.09	0
35	89.14	86.7	216	B6TQ08	Actin-1	2.12 ± 0.13	0
36	85.99	87.8	204	C0HDZ6	Actin-1	2.84 ± 0.38	0
37	33.75	59.0	23	B6TK17	DREPP2 protein	2.49 ± 0.34	0
38	25.77	48.8	23	B6TNI6	DREPP4 protein	2.25 ± 0.18	0
39	16.97	41.2	12	B6UAB6	Beta-expansin 1a	2.63 ± 0.15	0
Redox and defense							
40	28.82	51.1	24	B4FBH0	Peroxidase	2.34 ± 0.50	0
41	24.22	36.2	16	B4FY83	Peroxidase	2.62 ± 0.18	1
42	23.85	42.5	14	B4G0X5	Peroxidase	1.82 ± 0.09	1
43	12.61	19.2	7	B6STZ5	Peroxidase	2.03 ± 0.33	1
44	21.91	23.6	12	B4FA21	Purple acid phosphatase	2.13 ± 0.28	1
45	8.31	16.2	6	B4FM74	Purple acid phosphatase	3.57 ± 0.36	1
46	26.06	54.4	27	B6SWS9	Purple acid phosphatase	1.94 ± 0.14	0
47	20.19	34.1	16	B6TE04	Purple acid phosphatase	2.44 ± 0.43	1
48	40.99	60.1	27	B4F9H1	Glycosyltransferase	2.80 ± 0.31	0
49	44.62	61.0	35	B4FQW1	Glycosyltransferase	1.85 ± 0.33	0
50	6.98	11.7	5	C0P5K5	Glycosyltransferase	2.68 ± 0.32	0
51	53.56	61.3	60	B6UBI2	4-coumarate--CoA ligase	1.86 ± 0.11	0
52	49.82	59.0	61	H2BJB6	4-coumarate--CoA ligase	1.61 ± 0.13	0

Red and green represent increased and decreased in protein abundance in response to low K stress. The number of transmembrane helices predicted by TMHMM (version 2.0).

**Table 2:** List of the DAPs of K inefficient maize genotype in response to low K stress.

No.	Protscore	Coverage (95%)	Peptides (95%)	Accession no.	Proteins name	Fold change	THMs
Transportation							
1	6.05	7	4	W5U4T4	Potassium transporter	3.00 ± 0.33	13
2	30.12	57.6	32	B4FDN9	V-type proton ATPase subunit D	2.34 ± 0.12	0
3	28.11	31.2	17	C0PEG0	V-type proton ATPase subunit H	1.95 ± 0.15	0
4	8.8	12.5	6	B4FS42	Aquaporin PIP2-6	3.47 ± 0.34	7
5	9.78	4	7	K7VCA6	ABC transporter C family member 4	2.96 ± 0.28	15
6	11.68	18.9	15	C0HG48	Transmembrane 9 superfamily member	1.53 ± 0.11	10
7	50.42	50.2	35	C0P406	Vesicle-fusing ATPase	2.07 ± 0.10	0
8	43.89	76.1	41	B6TBM1	Alpha-soluble NSF attachment protein	2.03 ± 0.16	0
Signal transduction							
9	3.22	3.1	5	A0A096QE31	non-specific serine/threonine protein kinase	3.10 ± 0.19	2
10	31.5	75.4	23	B4FAE1	Ras-related protein RABD2c	1.51 ± 0.21	0
11	30.38	67.3	17	B4FRS1	Ras-related protein RABH1b	2.15 ± 0.13	0
12	31.9	73.7	21	P49103	Ras-related protein Rab-2-A	2.70 ± 0.18	0
13	45.26	78.5	48	B6SRI4	14-3-3-like protein	1.89 ± 0.16	0
14	39.04	72.6	36	B6TFS9	14-3-3-like protein A	1.87 ± 0.11	0
15	45.24	81	41	K7U5A5	14-3-3-like protein	2.19 ± 0.18	0
16	12.14	27.3	7	B6T460	Gibberellin receptor GID1L2	1.67 ± 0.14	0
17	28.14	29.4	18	B6TKK9	IQ calmodulin-binding motif family protein	1.73 ± 0.20	0
18	20.88	28.7	18	B4G0H5	Membrane-associated salt-inducible protein like	1.72 ± 0.10	0
19	3.07	7.5	2	B4FV79	Suppressor/enhancer of lin-12 protein 9	1.83 ± 0.11	1
20	29.7	93.9	26	B6T4U8	Calmodulin	2.64 ± 0.22	0
21	46.84	66.2	62	B4F9K2	Calcium-dependent lipid-binding	1.64 ± 0.18	1
22	9.78	19	5	B4FGP1	Calcium-dependent lipid-binding (CaLB domain) family protein	1.59 ± 0.15	0
23	24.66	41.5	13	B6TPQ1	Calcium-dependent phosphotriesterase superfamily protein	1.94 ± 0.17	1
24	26.12	56.7	14	B4FUC4	Plasma membrane-associated cation-binding protein 1	2.39 ± 0.13	0
25	50.68	80	53	B6U990	Ankyrin repeat domain-containing protein 2	1.79 ± 0.26	0
Lipid metabolism							
26	59.82	38.2	44	C0HFU7	Phospholipase D	3.49 ± 0.36	0
27	71.7	45	61	K7VQG5	Phospholipase D	2.34 ± 0.16	0
28	78.34	81.5	100	B4FHT1	Annexin	1.53 ± 0.12	0
29	59.83	45.2	36	B7ZZ50	Lipoxygenase	1.65 ± 0.11	0
30	191.05	81.9	278	Q9LKL4	Lipoxygenase	1.66 ± 0.22	0
31	109.13	71.3	115	Q8W0V2	linoleate 9S-lipoxygenase	5.22 ± 0.74	0
32	17.59	35.4	9	B6TAD3	Phospholipid scramblase	2.15 ± 0.24	0
33	15.83	33.7	10	Q49HE2	12-oxo-phytodienoic acid reductase	2.19 ± 0.34	0
Cellular structure							
34	9.19	26.1	8	K7VCH0	Expansin-B4	1.96 ± 0.12	1
35	16.44	31.2	11	B4FPG3	Hydroxyproline-rich glycoprotein family protein	1.89 ± 0.15	0
36	14.13	27.6	9	C0PD01	Fasciclin-like arabinogalactan protein 10	1.71 ± 0.13	0
37	3.84	22.5	6	B4FGH8	Non-classical arabinogalactan protein 31	1.89 ± 0.12	1
38	66.83	18	37	K7VNZ6	Protein MOR1	2.46 ± 0.21	0
39	30.06	77.6	34	K7W544	Putative carboxylesterase 2	2.39 ± 0.25	0
40	33.75	59	23	B6TK17	DREPP2 protein	2.98 ± 0.11	0
41	25.77	48.8	23	B6TNI6	DREPP4 protein	4.90 ± 0.65	0
42	16.97	41.2	12	B6UAB6	Beta-expansin 1a	3.09 ± 0.50	0
Redox and defense							
43	34.85	48.2	32	B4FSW5	Peroxidase	1.53 ± 0.13	0
44	32.58	67.3	57	B6T3V1	Peroxidase	2.89 ± 0.38	1
45	21.91	23.6	12	B4FA21	Purple acid phosphatase	2.16 ± 0.19	1
46	16.72	32.1	14	B6TE04	Purple acid phosphatase	1.53 ± 0.14	1
47	3.81	3.1	2	B7ZYZ7	Glycosyltransferase	2.69 ± 0.25	0
48	54.12	56	33	K7VBT8	4-coumarate--CoA ligase	1.60 ± 0.08	0
49	37.19	41.7	23	B4G058	4-coumarate--CoA ligase	2.69 ± 0.21	2

Red and green represent increased and decreased in protein abundance in response to low K stress. The number of transmembrane helices predicted by TMHMM (version 2.0).

**Figure 6:**
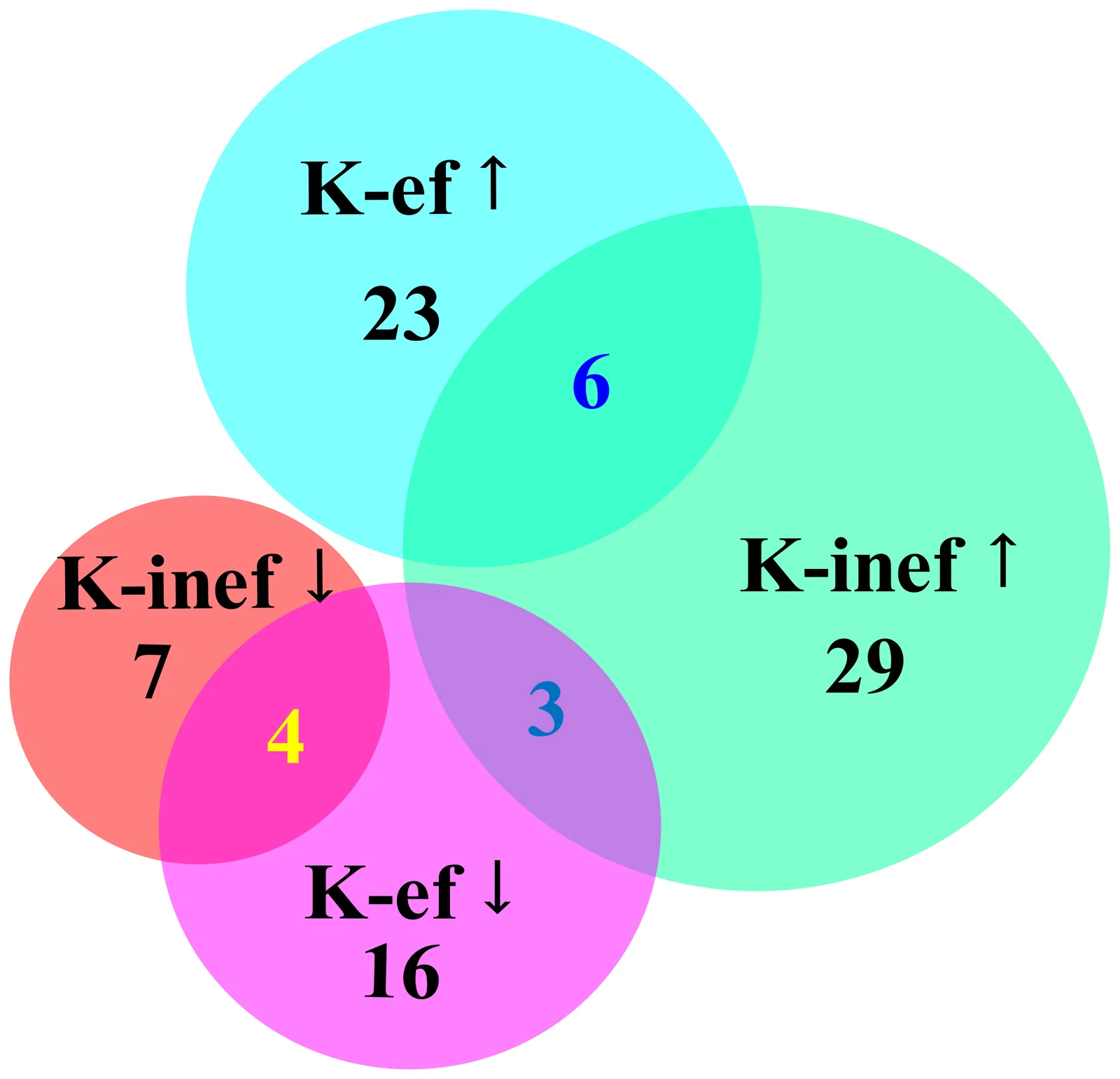
The K-ef unique, K-inef unique and commonly identified DAPs of PM (overlap area) between K-ef and K-inef. Up arrow and down arrow represent up- and down-regulated DAPs under low K stress, respectively.

**Figure 7:**
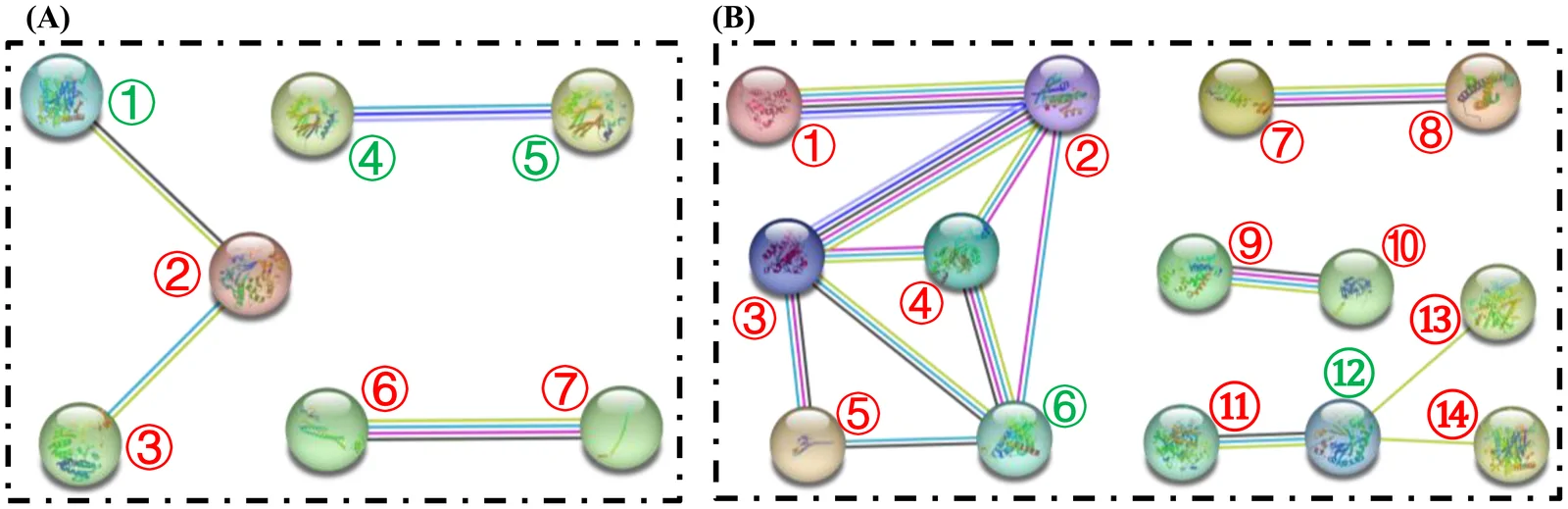
Protein interaction network of identified PM DAPs in K-ef **(A)** and K-inef **(B)** in response to low K stress. The network was built using a STRING software (https://string-db.org/) with medium confidence. The nodes represent the DAP, and the line color indicates the type of interaction evidence. The red and green represent the DAP increased and decreased in protein abundance, respectively. The DAPs name of **(A)** as follow: ①Lipoxygenase, ②12-oxo-phytodienoic acid reductase, ③4-coumarate--CoA ligase, ④V-type proton ATPase subunit C, ⑤V-type proton ATPase subunit G, ⑥Purple acid phosphatase, ⑦Purple acid phosphatase; The DAPs name of **(B)** as follow: ①Ras-related protein Rab-2-A, ②Ras-related protein RABH1b, ③Ras-related protein RABD2c, ④Vesicle-fusing ATPase, ⑤Suppressor/enhancer of lin-12 protein 9, ⑥Alpha-soluble NSF attachment protein, ⑦V-type proton ATPase subunit H, ⑧V-type proton ATPase subunit D, ⑨Peroxidase, ⑩Fasciclin-like arabinogalactan protein 10, ⑪4-coumarate--CoA ligase, ⑫12-oxo-phytodienoic acid reductase, ⑬Lipoxygenase, ⑭Lipoxygenase. The number with red and green represent increased and decreased in proteins abundance, respectively.

KEGG pathway enrichment was conducted based on the identified DAPs. The PM DAPs of K-ef were significantly enriched in 7 pathways (p < 0.05), including linoleic acid metabolism, metabolic pathways, phenylpropanoid biosynthesis, biosynthesis of secondary metabolites, phosphatidylinositol signaling system and riboflavin metabolism (**Table 3**). In contrast, PM DAPs of K-inef were significantly enriched in only one pathway, linoleic acid metabolism (p < 0.05) (**Table 3**). These results indicate that the K-efficient genotype (K-ef) activated a broader and more diverse repertoire of metabolic and signaling pathways under low K stress, enabling a multi-level coordinated response. In comparison, the response of the K-inefficient genotype (K-inef) was largely confined to linoleic acid metabolism, which may contribute to its reduced capacity to adapt to K deficiency.

**Table 3:** KEGG pathway enriched based on the PM DAPs.

No.	Pathway ID	Term description	Number of DAPs			
			K-ef↑	K-ef↓	K-inef↑	K-inef↓
1	zma00591	Linoleic acid metabolism	0	2	3	0
2	zma01100	Metabolic pathways	9	5	0	0
3	zma00592	alpha-Linolenic acid metabolism	1	2	0	0
4	zma00940	Phenylpropanoid biosynthesis	4	0	0	0
5	zma01110	Biosynthesis of secondary metabolites	5	3	0	0
6	zma04070	Phosphatidylinositol signaling system	2	1	0	0
7	zma00740	Riboflavin metabolism	2	0	0	0

The up and down arrow behind the cultivars represent increased and decreased in protein abundance under low K stress, respectively.

## 4 Discussion

Low K stress imposed a significant challenge to plants by disrupting cellular ionic homeostasis and impairing multiple physiological processes. The PM serves as the primary interface between the cell and the external environment. In the present study, comparative proteomic analysis of root PM protein from two maize genotypes with contrasting K efficiency revealed distinct regulatory patterns in response to low K stress, highlighting genotype-specific molecular strategies for adaptation to K deprivation.

### 4.1 Low K stress affects PM proteins in transportation

The dynamic regulation of PM transport proteins is critical for plant adaptation to K deficiency. A potassium transporter protein was significantly induced by low K stress in both maize genotypes, and the increased protein abundance of K-ef (increased 18 times) was significantly higher than that of K-inef (increased 3 times) (**Table 1**; **Table 2**). Consistent with this finding, the K concentration and K accumulation of K efficient maize (K-ef) were higher than that of K inefficient maize (K-inef) (**Figure 2**) suggesting that enhanced accumulation of the potassium transporter contributes to the superior K uptake capacity of the K-efficient genotype. In addition, Syntaxin 23, a member of the SNARE (soluble N-ethylmaleimide-sensitive factor protein attachment protein receptor) family involved in vesicle trafficking was also increased in K-ef but not in K-inef (**Table 1**). Although the functional role of Syntaxin 23 in K transport remains unclear, its homolog Syntaxin 121 has been shown to physically interact with K channels to facilitate K uptake in Arabidopsis (Lefoulon et al., 2018). Previous studies indicated that plants possessed multiple K transporter families which are induced by low K stress, especially the high-affinity K transporters (Wang and Wu, 2017; Cui et al., 2025). Patellin proteins are putative regulators of membrane trafficking in plants (Zhou et al., 2019). The decreased abundance of patellin1 in K-ef may reflect a reorganization of membrane trafficking pathways that preferentially allocates cellular resources toward the synthesis, trafficking, or plasma membrane localization of K transporters, thereby enhancing K acquisition under K-deficient conditions.

In K inefficient maize genotype (K-inef), a greater number of PM proteins were increased in protein abundance under low K stress. Notably, V-type proton ATPase subunits D and H increased abundance in K-inef (**Table 2**), in contrast to the decreased abundance of V-type proton ATPase subunits G and C in K-ef (**Table 1**). V-ATPase plays an essential role in energizing secondary active transport and is indispensable for plant growth and development. V-ATPase consists of two functional domains (V0 and V1) formed by at least 13 different subunits. The V1 domain is composed of eight subunits (A to H) and is responsible for ATP hydrolysis, providing the energy required for proton transport across membranes (Wang C et al., 2021). Similarly, ATP-binding cassette (ABC) transporters are primary active transporters that directly utilize ATP hydrolysis to drive substrate transport across membranes. Therefore, both V-ATPase and ABC transporters consume ATP. The increased abundance of ABC transporter C family member 4 and V-type proton ATPase subunits in K-inef genotype created a dual drain on cellular ATP reserves, as both transport systems are highly energy dependent. In contrast, K-ef genotype decreased the abundance of several energy-intensive transport systems while preferentially allocating its limited ATP resources specifically in K transport and essential trafficking components. Accordingly, K-inef exhibited a markedly different pattern of protein regulation, with a broader and more diverse set of increased proteins under K deficiency. This contrasting pattern of protein regulation is consistent with the physiological performance of the two genotypes. Under K-deficient conditions, K-ef genotype displayed a greater root K uptake capacity (**Figure 5**), higher K concentration, and greater K accumulation (**Figure 2**) than K-inef genotype. Root vitality of K-ef was significantly lower than K-inef under high K, but significantly higher under low-K stress (**Figure 4**). The K efficient soybean genotype also had a lower root vitality compared to the K inefficient soybean genotype under high K condition (Liu et al., 2021). These findings suggest that the K-efficient maize genotype (K-ef) might adopt an “energy-saving” strategy under low K stress, focusing limited ATP resources on K transport and essential trafficking components, whereas the K-inefficient genotype (K-inef) fallen into a “widespread stress response, energy-wasting” pattern that ultimately hindered efficient K acquisition.

### 4.2 Low K stress affects PM proteins in signal transduction

Calcium signaling plays a central role in plant responses to K deprivation by coordinating the activation of K uptake and adaptive stress responses (Wang et al., 2018). In the K-ef genotype, 5 calcium-related proteins were significantly increased in protein abundance under low K stress (**Table 1**). In contrast, calmodulin and multiple calcium-dependent lipid-binding proteins (CaLB domain family protein and calcium-dependent phosphotriesterase superfamily protein), as well as plasma membrane-localized Ca^2+^-binding protein (plasma membrane-associated cation-binding protein 1) were decreased in protein abundance in K-inef (**Table 2**). Calmodulin and calmodulin-like protein are primary Ca^2+^ sensors that transduce signals by interacting with and modulating various target proteins, including transporters, kinases, and transcription factors. The Ca^2+^ signaling was activated by K deficiency, which promotes K absorption by regulating the expression of K channel genes (NtCNGC3 and NtCNGC10) and transporter genes in tobacco root (Wang Y. et al., 2021). Similarly, in maize, calcium signaling pathway promoted K absorption and the expression of K uptake-related genes in roots under low K stress (Xia et al., 2023). Consistent with these findings, the K-efficient genotype (K-ef) exhibited greater root K uptake capacity (**Figure 5**) and higher K accumulation under K deficiency (**Figure 2**). Moreover, increased protein abundance of calmodulin and calmodulin-like protein suggested an enhanced capacity for Ca^2+^-dependent signaling under low K stress, which might facilitate the activation of K acquisition mechanisms in K-ef.

In addition, some PM proteins showed a decrease in protein abundance in K-ef but increased in K-inef. Specifically, these PM proteins were 14-3–3 protein and suppressor/enhancer of lin-12 protein 9 (**Table 1** and **Table 2**). Previous studies have shown that 14-3–3 proteins regulate K channels and are involved in K deficiency signaling (Sottocornola et al., 2008; Xu and Shi, 2006). Moreover, 14-3–3 proteins interact with multiple target proteins to participate in the adaptation of plants to nutrient deficiency (Shin et al., 2011). Comparative proteomics in Arabidopsis identified 14-3–3 as a candidate in K deficiency signaling pathways (Kang et al., 2004). The opposite regulation of these signaling proteins indicated a fundamental difference in signal integration between the two maize genotypes. The K-efficient maize genotype (K-ef) might down-regulate certain 14-3–3 isoforms to reallocate cellular resources toward core K^+^-uptake machinery and to relieve constraints on K channel activity. These findings highlight that the ability to precisely reprogram signal transduction networks rather than simply activating stress signaling is a key determinant of K utilization efficiency in maize.

### 4.3 Low K stress affects PM proteins in lipid metabolism

Lipid metabolism is intimately linked to the structural integrity and functional dynamics of the PM, particularly under abiotic stress conditions. 12-Oxo-phytodienoic acid reductases (OPRs) are the key precursors in the biosynthesis of jasmonic acid (Li et al., 2009). Lipoxygenases (LOXs) catalyze the dioxygenation of polyunsaturated fatty acids (linoleic and linolenic acids) to generate fatty acid hydroperoxides, which are precursors for the synthesis of jasmonic acid and other oxylipins involved in stress responses (Singh et al., 2022). The OPR was increased in K-efficient maize genotype (K-ef) but decreased in K-inefficient maize genotype (K-inef) in response to low K stress. In contrast, two LOXs were decreased in K-ef which were increased in K-inef (**Table 1** and **Table 2**). By enhancing OPR activity while suppressing LOX, K-ef may limit the production of reactive oxylipins that could cause membrane damage. The increased LOX in K-inef might also contribute to lipid peroxidation and membrane deterioration, exacerbating the effects of K deficiency.

Phospholipase D hydrolyzes membrane phospholipids to produce phosphatidic acid and a free head group. As an important lipid signaling molecule, phosphatidic acid can activate NADPH oxidases thereby promoting reactive oxygen species (ROS) production, and can also regulate the activity of ion channels, transporters, and other signaling proteins (Hafsi et al., 2009). Phospholipid scramblase catalyzes the bidirectional translocation of phospholipids across the plasma membrane, disrupting the normal asymmetric distribution of lipids (Pandey et al., 2025). In the present study, both phospholipase D and phospholipid scramblase were increased in K-inef (**Table 2**) but not in K-ef. K-efficient maize genotype (K-ef) adopted a “restrained” lipid metabolism strategy under low K stress by decreasing the LOX and annexins and increasing OPR (**Table 1**). In contrast, K-inefficient maize genotype (K-inef) exhibited an “overactive” or “dysregulated” lipid metabolism response by increasing LOX and decreasing OPR (**Table 2**).

### 4.4 Low K stress affects PM proteins in redox and defense

Low K stress imposes secondary oxidative stress on plants, as K deficiency impairs multiple metabolic processes, including photosynthetic electron transport, ion homeostasis and cellular redox balance. To minimize oxidative damage and preserve plasma membrane integrity, plants activate a suite of defense-related proteins to manage this process. In the present study, there were 13 PM proteins in K-ef compared to 7 PM proteins in K-inef that increased in protein abundance in response to low K stress (**Table 1** and **Table 2**). Plasma membrane- and cell wall-localized peroxidases are particularly important for controlling apoplastic ROS (reactive oxygen species) levels, which can otherwise trigger oxidative damage to membrane lipids and proteins, as well as impair transporter function. In this study, there were four peroxidases in K-ef and two peroxidases in K-inef increased in protein abundance in response to low K stress (**Table 1** and **Table 2**). Previous studies have indicated that the K-sensitive maize suffered seriously damaged than the K-tolerant maize by ROS under K deficiency (Du et al., 2019). Low K stress has been extensively documented to trigger significant changes in redox- and defense-related proteins across diverse plant species. In wild soybean, the enhanced ROS scavenging capacity was a key factor contributing to low K tolerance (Li et al., 2025). Similarly, in barley, proteomic analyses identified 129 defense-related DAPs that distinguish tolerant from sensitive genotypes, with ethylene and phenylpropanoid pathways playing pivotal roles (Zeng et al., 2015). Therefore, the redox and defense proteome undergoes substantial remodeling under low- K stress.

Glycosyltransferases (GTs) play essential roles in cell wall biosynthesis, protein glycosylation (which affects protein stability and trafficking), and the glycosylation of secondary metabolites. Under low K stress, glycosylation could enhance the solubility and activity of defense compounds and protect membranes from oxidative damage. In the present study, the multiple GTs increased in K-ef might reflect enhanced cell wall remodeling and reinforcement under low K stress, as well as improved glycosylation of PM proteins to ensure their proper folding, stability, and membrane localization. In addition, GT-mediated glycosylation of small molecule antioxidants may increase their ROS-scavenging capacity. Collectively, the stronger induction of antioxidant and cell wall-associated enzymes in the efficient maize genotype (K-ef) likely enables better control of apoplastic ROS levels, preservation of PM integrity, and maintenance of transporter function under low K stress.

## 5 Conclusion

Physiological and PM proteomic responses of two maize genotypes with contrasting K efficiency under low K stress were comprehensively compared in the present study. K-efficient maize genotype (K-ef) coped with low K stress through a coordinated, energy-conserving, and multi-pathway strategy at the PM level that integrated transport, signal transduction (particularly Ca^2+^ and 14-3-3 modulation), lipid metabolism, and redox/defense systems. In contrast, the K-inefficient maize genotype (K-inef) suffered from indiscriminate protein up-regulation and insufficient activation of core K-uptake components. These findings provide a valuable proteomic resource and novel targets for improving K tolerance in maize and other crops through genetic or biotechnological approaches.

## Data Availability

The original contributions presented in the study are included in the article/**Supplementary Material**. Further inquiries can be directed to the corresponding author/s.
